# Fluorescence-Based Detection of Natural Transformation in Drug-Resistant Acinetobacter baumannii

**DOI:** 10.1128/JB.00181-18

**Published:** 2018-09-10

**Authors:** Anne-Sophie Godeux, Agnese Lupo, Marisa Haenni, Simon Guette-Marquet, Gottfried Wilharm, Maria-Halima Laaberki, Xavier Charpentier

**Affiliations:** a Centre International de Recherche en Infectiologie, INSERM U1111, Université Claude Bernard Lyon 1, CNRS, UMR5308, École Normale Supérieure de Lyon, Université de Lyon, Villeurbanne, France; b Université de Lyon, VetAgro Sup, Marcy l'Etoile, France; c Unité Antibiorésistance et Virulence Bactériennes, Université Claude Bernard Lyon 1, ANSES Site de Lyon, Lyon, France; d Robert Koch Institute, Wernigerode Branch, Wernigerode, Germany; Michigan State University

**Keywords:** acinetobacter, Acinetobacter baumannii, cytometry, horizontal gene transfer, natural transformation

## Abstract

Acinetobacter baumannii is a nosocomial agent with a high propensity for developing resistance to antibiotics. This ability relies on horizontal gene transfer mechanisms occurring in the Acinetobacter genus, including natural transformation. To study natural transformation in bacteria, the most prevalent method uses selection for the acquisition of an antibiotic resistance marker in a target chromosomal locus by the recipient cell. Most clinical isolates of A. baumannii are resistant to multiple antibiotics, limiting the use of such selection-based methods. Here, we report the development of a phenotypic and selection-free method based on flow cytometry to detect transformation events in multidrug-resistant (MDR) clinical A. baumannii isolates. To this end, we engineered a translational fusion between the abundant and conserved A. baumannii nucleoprotein (HU) and the superfolder green fluorescent protein (sfGFP). The new method was benchmarked against the conventional antibiotic selection-based method. Using this new method, we investigated several parameters affecting transformation efficiencies and identified conditions of transformability one hundred times higher than those previously reported. Using optimized transformation conditions, we probed natural transformation in a set of MDR clinical and nonclinical animal A. baumannii isolates. Regardless of their origin, the majority of the isolates displayed natural transformability, indicative of a conserved trait in the species. Overall, this new method and optimized protocol will greatly facilitate the study of natural transformation in the opportunistic pathogen A. baumannii.

**IMPORTANCE** Antibiotic resistance is a pressing global health concern with the rise of multiple and panresistant pathogens. The rapid and unfailing resistance to multiple antibiotics of the nosocomial agent Acinetobacter baumannii, notably to carbapenems, prompt to understand the mechanisms behind acquisition of new antibiotic resistance genes. Natural transformation, one of the horizontal gene transfer mechanisms in bacteria, was only recently described in A. baumannii and could explain its ability to acquire resistance genes. We developed a reliable method to probe and study natural transformation mechanism in A. baumannii. More broadly, this new method based on flow cytometry will allow experimental detection and quantification of horizontal gene transfer events in multidrug-resistant A. baumannii.

## Abstract (precis)

Antibiotic resistance is a pressing global health concern with the rise of multiple and panresistant pathogens. The rapid and unfailing resistance to multiple antibiotics of the nosocomial agent Acinetobacter baumannii, notably to carbapenems, prompt to understand the mechanisms behind acquisition of new antibiotic resistance genes. Natural transformation, one of the horizontal gene transfer mechanisms in bacteria, was only recently described in A. baumannii and could explain its ability to acquire resistance genes. We developed a reliable method to probe and study natural transformation mechanism in A. baumannii. More broadly, this new method based on flow cytometry will allow experimental detection and quantification of horizontal gene transfer events in multidrug-resistant A. baumannii.

## INTRODUCTION

Acinetobacter baumannii is a Gram-negative bacterium responsible for health care-associated infections in humans and animals (1, 2). Outside the hospital, it has been detected in various environments (3), but its exact reservoir remains unclear. Asymptomatic carriage of A. baumannii has been reported in humans and animals, although the prevalence in healthy individuals seems low (4–7). Although rarely encountered in animals, Acinetobacter infections represent a major challenge for physicians in intensive care units since acquired antibiotic resistance is widespread in A. baumannii isolates. Worldwide, the percentage of invasive isolates with combined resistance to fluoroquinolones, aminoglycosides, and carbapenems is on the rise (8, 9). The ability of this bacterium to acquire multiple antibiotic resistance genes is well established, but the underlying mechanisms are not clearly understood. Genome sequencing of multidrug-resistant (MDR) strains revealed that multiple events of horizontal gene transfer are responsible for the acquired multidrug resistance (10, 11) and that extensive recombination events drive the diversification of A. baumannii, resulting also in antigenic variation (12). Among the horizontal gene transfer mechanisms, natural transformation has been demonstrated in some A. baumannii isolates, providing a plausible route for intra- and interspecific genetic exchanges (13–15). Natural transformation could notably explain the frequent occurrence in A. baumannii genomes of large genomic islands lacking features characteristic of self-transmissible elements, such as the AbaR genomic islands (16, 17). These islands carry multiple resistance genes and have also been revealed in pathogenic non-baumannii species (18). Natural transformation allows a bacterial cell to take up exogenous DNA and subsequently incorporate it into its genome through homologous recombination (19). Although some sequence identity is required between the donor DNA and the recipient's chromosome for recombination, natural transformation allows integration of more diverse sequences such as transposons, integrons, and gene cassettes from distant species (20). The recombination of exogenous DNA into the chromosome requires that the bacteria first enter the physiological state of competence, which includes the expression of a molecular machinery to take up DNA, protect it from degradation, and bring it to the chromosome. Although natural transformation is a conserved trait in bacteria, the conditions required to trigger competence and to transform are often elusive and species specific (19, 21). Interestingly, some antibiotics were found to induce competence in three distinct human pathogens, namely, Helicobacter pylori, Legionella pneumophila, and Streptococcus pneumoniae, raising the concern that some antibiotic treatment may increase horizontal gene transfer events (22–24). More specifically in A. baumannii and related species, the description of natural transformation is rather recent and occurs upon movement on wet surfaces (13, 15).

Natural genetic transformation is generally detected through a phenotypic outcome. Acquisition of antibiotic resistance conferred to the cells by the chromosomal integration of a provided DNA fragment carrying an antibiotic resistance gene represents the most common phenotypic outcome (13). However, since most clinical isolates of A. baumannii are resistant to multiple antibiotics, using antibiotic selection to evaluate natural transformation in A. baumannii appears both experimentally challenging and ethically questionable. Thus, an alternative method to detect and quantify transformation events is needed to explore natural transformation in MDR clinical A. baumannii isolates. Previous work in the related species Acinetobacter baylyi indicated that green fluorescent protein (GFP) could be used as a selection-free method to qualitatively assess natural transformation in this nonpathogenic species (25). We present here a phenotypic and selection-free method based on flow cytometry to quantitatively assess transformation in A. baumannii. To this end, we developed a flow cytometry-optimized fluorescent marker consisting of a chromosomal translational protein fusion between a nucleoid-associated protein (HU) and the superfolder GFP (sfGFP). Flow cytometry effectively discriminates HU-sfGFP green fluorescent A. baumannii cells among a large population of nonfluorescent cells. We demonstrated that DNA encoding the HU-sfGFP marker is a suitable substrate for genetic transformation in A. baumannii. Compared to selection-based detection using an antibiotic resistance marker, flow cytometry combined with the HU-sfGFP marker offers a more reliable and direct quantification of transformants. We took advantage of this transformation detection method to improve transformation conditions for A. baumannii and to probe natural transformation in MDR clinical isolates but also in nonclinical strains.

## RESULTS

### Optimization of a bright chromosomally encoded GFP fusion in A. baumannii.

**FIG 1:**
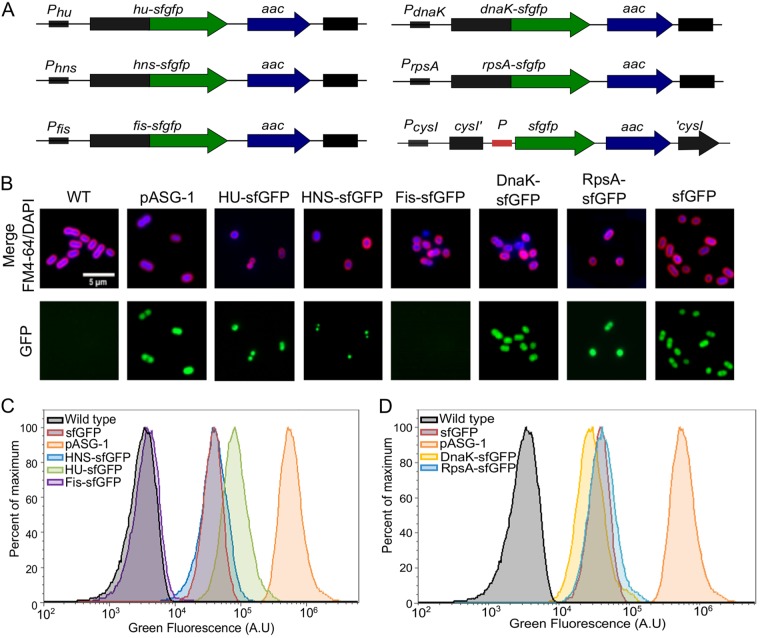
Engineering a chromosomal fluorescent marker in A. baumannii. (A) Schematic representation of genetic constructions encoding the fluorescent markers used in this study. The gene encoding sfGFP and sfGFP fused to the C terminus of HU, HNS, Fis, DnaK, and RpsA proteins is represented in green. An *aac*(*3*)*IV* gene encoding resistance to apramycin is indicated in blue. The red box corresponds to a synthetic constitutive promoter. The sequences and genes in black are part of the AB5075 chromosome. (B) Fluorescence microscopy images of A. baumannii AB5075 derivatives expressing free or chimeric sfGFP proteins. Membranes were stained with FM4-64 and DNA with DAPI (upper row and first column). For illustration purposes, the green fluorescence intensities were normalized between the samples (lower row). (C and D) Flow cytometry histograms of the green fluorescence intensities of AB5075 derivatives expressing free sfGFP or sfGFP protein fusions in comparison to the wild-type level (WT). The results from a representative experiment are shown.

We sought to use expression of GFP as a fluorescence-based phenotypic outcome of natural transformation. To allow the distinction of bacterial transformants from a larger nonfluorescent bacterial population, an optimal fluorescent marker should therefore confer a fluorescence bright enough to be discriminated from bacterial autofluorescence. Moreover, in order to be a suitable substrate for natural transformation in various A. baumannii isolates, this marker must insert in a conserved locus in the A. baumannii chromosome. We first took advantage of the recent strategy developed by Kjos et al., who engineered a bright strain of S. pneumoniae using a translational C-terminal fusion of the superfolder GFP (sfGFP) to HlpA, the unique nucleoid-associated protein of this Gram-positive pathogen (26). In Gram-negative bacteria, 12 nucleoid-associated proteins (NAPs) that possess DNA-binding activity are commonly described (27). Using the BLAST algorithm, we searched for genes encoding nucleoid-associated proteins in the genome of the multidrug-resistant clinical isolate A. baumannii AB5075. We identified three homodimeric small NAPs: HU (ABUW_2198), HNS (ABUW_3609), and Fis (ABUW_1533). Two other candidate proteins were also selected based on their abundance inferred from proteomic analysis performed in A. baumannii strain ATCC 17978 (28). In this particular strain, heat shock protein DnaK (ABUW_3879) and ribosomal protein S1 RpsA (ABUW_2242) were among the more abundant cytoplasmic proteins. Moreover, these two proteins appeared to be suitable candidates for GFP translation fusions based on successful C-terminal fusions in Mycobacterium smegmatis (29) and Bacillus subtilis (30). Consequently, we generated translational C-terminal fusions of the sfGFP with HU, HNS, Fis, DnaK, and RpsA proteins. Lastly, as a means of comparison, we constructed a strain carrying the sfGFP-encoding gene (referred to here as the sfGFP gene) under a strong constitutive synthetic promoter inserted into a neutral chromosomic locus in A. baumannii. The chosen locus was the sulfite reductase gene (ABUW_0643 or *cysI*) identified previously as a conserved but nonessential gene in A. baumannii (13). Using natural transformation, the six genetic constructs were independently integrated into the chromosome of the pathogenic AB5075 A. baumannii strain (Fig. 1A). A. baumannii was also transformed with a broad host-range and multicopy plasmid carrying the sfGFP gene under a strong constitutive promoter (plasmid pASG-1). Using Western blot analysis of bacterial lysates, we confirmed that all the AB5075 derivative strains expressed sfGFP protein fusions with the expected molecular weight (see Fig. S1 in the supplemental material). However, the Fis-sfGFP appeared to be less expressed than other sfGFP protein fusions. Then, fluorescence signals and subcellular localizations of the various sfGFP protein fusions were analyzed using fluorescence microscopy (Fig. 1B). Consistent with the immunoblotting analysis, the strain expressing the Fis-sfGFP marker presented a low green fluorescence that was comparable to the wild-type strain (autofluorescence, Fig. 1B). Otherwise, all other sfGFP fusions were detectable by fluorescence microscopy. Still, the cellular localization of the green fluorescence signal differs among the strains. As expected, free sfGFP expressed from either a chromosomal locus or from a plasmid (*cysI*::*sfgfp* or pASG-1) presented a diffuse localization pattern in bacterial cells. This localization pattern was similar for strains expressing sfGFP translation fusions with cytoplasmic proteins (DnaK-sfGFP and RpsA-sfGFP). In contrast, when sfGFP was fused to HU-sfGFP and HNS-sfGFP, the GFP signal showed a discrete pattern. The HU-sfGFP signal appeared as diffused as the nucleoid visualized by DAPI staining. Also, the subcellular localization of HNS-sfGFP was restricted to few discrete foci per cell. Subsequently, green fluorescence intensities of the various strains were then quantified by flow cytometry (Fig. 1C and D). All strains expressing sfGFP chromosomal markers, except Fis-sfGFP, exhibited a fluorescence distinct from the unmarked wild-type strain (autofluorescence). As expected, cytoplasmic sfGFP expressed from a replicative plasmid (pASG1) displayed a strong green fluorescence in comparison to the wild-type strain (200-fold when comparing the mean intensities), whereas the cytoplasmic sfGFP expressed from the chromosome presented an intermediate green fluorescence level (an ∼12-fold increase relative to autofluorescence). Among the chromosomal markers, the HU-sfGFP construct presented the strongest fluorescence with a mean of 20-fold increase compared to wild-type levels. The other genetic constructs (RpsA-sfGFP, DnaK-sfGFP, and HNS-sfGFP) presented fluorescence levels comparable to the free sfGFP expressed from the *cysI* locus. Remarkably, the distribution of the fluorescence intensity of population expressing HU-sfGFP protein fusion does not overlap the wild-type autofluorescence allowing a clear resolution of the two populations (Fig. 1C). We ensured that the various fluorescent strains presented a growth similar to the wild-type strain, indicating that both the translational fusions and their chromosomal insertions could be used for downstream applications (see Fig. S2A in the supplemental material). Moreover, all fluorescent markers but Fis-sfGFP appear to be steadily expressed during growth in liquid medium with, expectedly, HU-sfGFP presenting the strongest signal among the six chromosomal constructions (see Fig. S2B in the supplemental material). In conclusion, among the six chromosomal fluorescent markers investigated, expression of the HU-sfGFP marker confers the highest fluorescence level, and its expression is compatible with bacterial growth, both characteristics required to constitute a genetic marker of natural transformation.

### Benchmarking the HU-sfGFP chromosomal marker in A. baumannii transformation assay.

**FIG 2:**
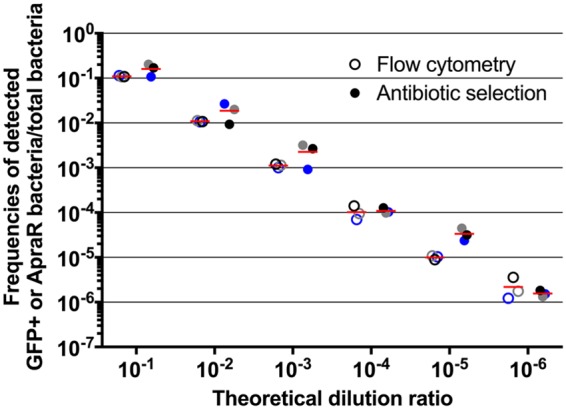
Comparison of paired measurements of *hu-sfgfp_aac* marker detection obtained by flow cytometry and antibiotic selection methods. HU-sfGFP cells were diluted into a suspension of wild-type A. baumannii AB5075 cells with increasing dilution factor (abscissa). Green fluorescent and apramycin-resistant populations were determined (ordinate) both by flow cytometry and antibiotic selection. The three dots represent technical replicates. For a given dilution ratio, dots of the same color indicate a sample tested by both methods; the horizontal lines represent the mean of the three measurements.

In an A. baumannii population, the frequency of transformants does not usually exceed 10^−3^, meaning that less than 1 of 1,000 bacterial cells integrate and express a selection marker (13). Thus, a fluorescence-based phenotypic outcome of natural transformation must be able to detect a rare population of sfGFP-expressing cells within a large population of nonfluorescent cells. Because of their high-resolution power, antibiotic resistance selection-based assays are frequently used to detect rare transformation events. We thus benchmarked the direct flow cytometry-based detection of the HU-sfGFP marker to the selection-based antibiotic marker detection classically used for testing natural transformation. To this end, the HU-sfGFP marker bears an apramycin resistance gene [*aac*(*3*)-*IV*], allowing comparison of both methods of selection/detection for the same chromosomal locus of recombination (Fig. 1A). First, we tested the sensitivity and specificity of flow cytometry-based detection of bacterial cells expressing the HU-sfGFP marker within large nonfluorescent bacterial populations and compared it to the apramycin resistance selection. We subjected to both methods of detection the same samples consisting of serial dilutions of HU-sfGFP cells into wild-type cells (Fig. 2). Both methods gave comparable determination of bacterial concentrations in the range of 10^−1^ to 10^−6^ dilution ratios and corresponded to the theoretical dilution factors. The latter dilution (10^−6^) represents the lower detection limit mostly for technical reasons. With our current equipment and settings, analysis of >10^6^ bacterial cells is possible but substantially increases the experimentation time.

**FIG 3:**
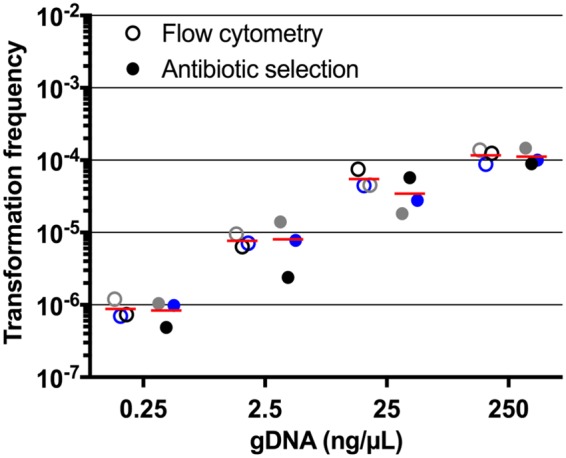
Comparison of flow cytometry sensitivity to antibiotic selection in detecting natural transformation with the *hu-sfgfp_aac* marker. Wild-type A. baumannii AB5075 cells were transformed using increasing amounts of *hu-sfgfp_aac* genomic DNA (gDNA). The DNA concentrations are expressed in nanograms of DNA per microliter of the bacterial suspension used in the transformation assay. The transformation frequencies were determined both by flow cytometry and by antibiotic selection for each gDNA concentration. For each method and DNA concentration, the three dots represent technical replicates. For a given gDNA concentration, dots of the same color indicate a sample tested by both methods. The horizontal lines represent the mean of the three measurements.

We then tested the sensitivity and specificity of flow cytometry for the detection of HU-sfGFP transformants within wild-type cells. To this end, we used the genomic DNA (gDNA) extracted from the *hu-sfgfp_aac* strain as the substrate for natural transformation of wild-type AB5075 strain and compared the frequency of transformants measured either by flow cytometry or by antibiotic selection (see Materials and Methods for a detailed description of natural transformation assay). In A. baylyi, transformation efficiency increases in a DNA concentration-dependent manner (31). To assess the accuracy and reliability of the cytometry-based method, we used increasing concentrations of DNA substrate for transformation experiments and found that both methods provided comparable results (Fig. 3). For both methods, transformation efficiencies were around 10^−6^ using 0.25 ng of *hu-sfgfp_aac* gDNA, and then the transformation frequencies increased by 10-fold with DNA concentration up to a transformation frequency of ∼10^−5^ for 2.5 ng of gDNA. However, for higher gDNA amounts (25 and 250 ng), the transformation frequencies seem to reach saturation and remained around 10^−4^. Altogether, these results demonstrate that detection of HU-sfGFP marker by flow cytometry constitutes a method as quantitative and specific as the classical antibiotic selection method to follow natural transformation in A. baumannii. The apramycin resistance cassette of the HU-sfGFP chromosomal marker was required here to benchmark the fluorescence-based method against the antibiotic selection method. However, our initial objective was to obtain a transformation marker that does not confer additional antibiotic resistance. We consequently engineered a HU-sfGFP chromosomal marker without an apramycin resistance cassette (see Fig. S3A in the supplemental material). We ensured that the new genetic construct was still compatible with bacterial growth (see Fig. S3B in the supplemental material) and that the fluorescence emitted was comparable to the *hu-sfgfp_aac* strain (see Fig. S3C in the supplemental material). Therefore, all of the following experiments were performed using the HU-sfGFP marker without any antibiotic resistance gene.

### Improving parameters of natural transformation of A. baumannii.

**FIG 4:**
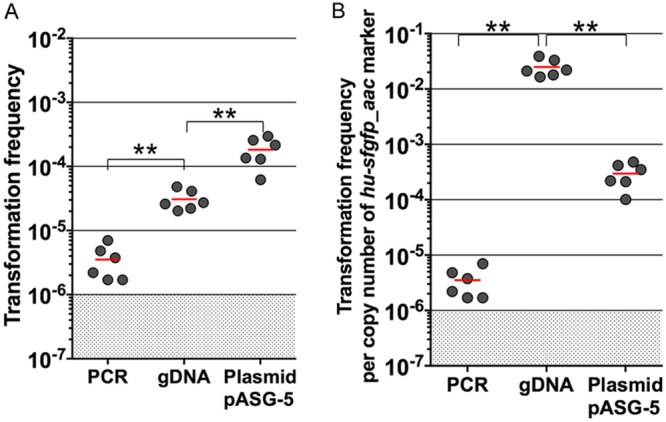
DNA forms and transformation efficiency. Comparison of transformation frequencies obtained with the *hu-sfgfp* marker on either a PCR fragment with 2 kb of flanking homology, gDNA, or a nonreplicative plasmid (pASG-5). The same mass of DNA (50 ng) was used for each DNA form. The graphics represent either absolute measurement (A) or results normalized to the copy number of the marker (B). A total of six independent transformation assays were performed on two separate occasions. The horizontal lines represent the means for each condition. The limit of detection (10^−6^) is indicated by a shaded area. Two-by-two comparison using the nonparametric Mann-Whitney-Wilcoxon test (two-tailed) between the various conditions yielded *P* values of <0.01 (**).

**FIG 5:**
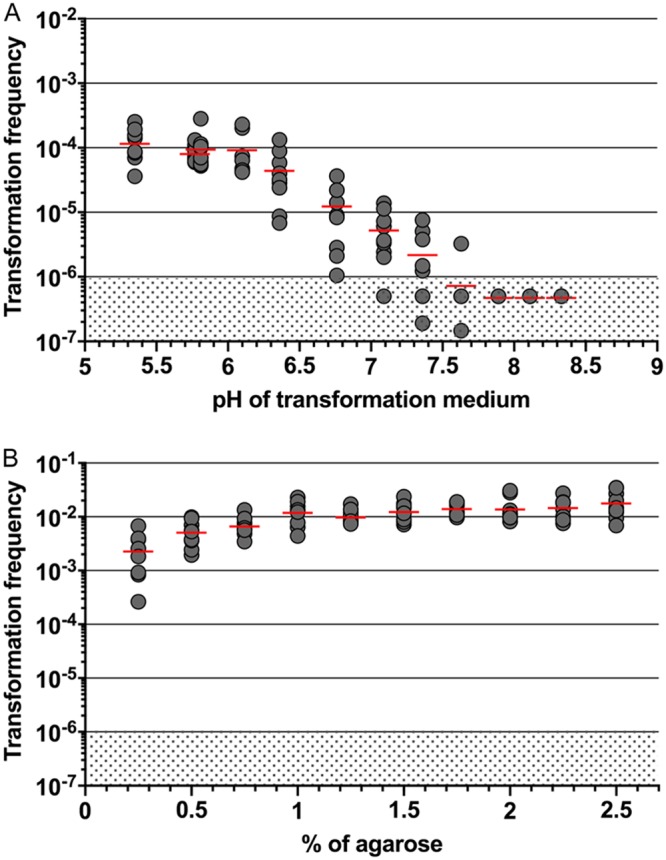
Parameters affecting transformability of A. baumannii. (A) Effect of pH on transformation efficiencies. Bacteria were transformed using 0.5% agarose in transformation medium buffered using potassium phosphate with a measured pH ranging from 5.35 to 8.33. The Spearman correlation coefficient between pH 5.35 and 6.1 and the transformation frequency is *r* = −0.4 (*P* [two tailed] > 0.05). The Spearman correlation coefficient between pH 6.1 and 7.89 and the transformation frequency is *r* = −1 (*P* [two tailed] < 0.01). (B) Effect of increasing concentrations of agarose on transformation efficiencies (0.5% agarose was used in the foregoing experiments). The Spearman correlation coefficient between agarose concentration and transformation frequency is *r* = 0.9758 (*P* [two tailed] < 0.0001). A total of nine independent transformation assays were performed on three separate occasions. The horizontal lines represent means for each condition. The limit of detection (10^−6^) is indicated by a shaded area.

To detect natural transformation in MDR clinical isolates, we first took advantage of the fluorescence-based method to optimize conditions of natural transformation in A. baumannii. Because less than a nanogram of gDNA already results in frequencies higher than 10^−6^, we set this value as the detection threshold of transformation. We first investigated how the type of transforming DNA would influence the transformation frequencies. A. baumannii strain AB5075 was subjected to transformation using the same mass of DNA (50 ng) carrying the HU-sfGFP marker flanked by 2-kb-long regions homologous to the targeted chromosomal locus, either as a linear PCR product, as an insertion in nonreplicative plasmid (pASG-5), or as integrated at its locus in gDNA (Fig. 4A). Transformation using gDNA resulted in transformation efficiencies between 10^−5^ and 10^−4^, which were comparable to the foregoing result (Fig. 3, 25 ng). However, transformation using a PCR product appeared less efficient, with at least 10-fold-fewer transformants than with gDNA. On the other hand, transformation frequencies using pASG-5 were significantly higher than using gDNA (mean of 1.5 × 10^−4^). As previously proposed for A. baylyi (31), the difference in transformation efficiency between gDNA and plasmid DNA (pASG-5) may be explained by the number of copies of markers, which was much greater when using plasmid DNA than in gDNA (Fig. 4B). With this result taken into consideration, the following experiments were performed using pASG-5 as the substrate of transformation. We tested first the impact of the bacterial culture and storage conditions prior to transformation assay and found that culturing bacteria in liquid broth results in slightly higher transformation efficiencies in comparison to bacteria stored at −80°C in conservation medium (5-fold decrease) (see Fig. S4 in the supplemental material). This decrease may be due partially to the presence of glycerol, given that the addition of glycerol to bacteria grown on solid or liquid media decreases transformation (respectively, by 4-fold and 2-fold) (see Fig. S4 in the supplemental material). The optimal pH values for natural transformation have been reported to be more than 6.5 for A. baylyi ADP1 and 7.5 in A. baumannii A118 (31, 32). We therefore investigated the effect of pH on natural transformation in strain AB5075. To this end, transformation medium was buffered in the range of 5.3 to 8.3 using the potassium phosphate buffering system of Palmen et al. (31) (Fig. 5A). We observed that at between pH 5.3 and pH 6.1, the transformation levels were around 10^−4^. For pH values greater than 6.36, the transformation frequencies decrease by 10-fold steadily down to 100-fold for a pH above 7.36 to reach the lower detection limit. Based on the study of Traglia et al. performed on the A. baumannii strain A118, we also investigated the role of bovine serum albumin (BSA) on transformation efficiency and found no concentration-dependent effect on transformation level when 0.25 to 1% BSA was added to the transformation medium (see Fig. S5A in the supplemental material). On the contrary, the addition of BSA results in a moderate decrease in transformation (3- to 4-fold). We also investigated the role of divalent cations (Ca^2+^, Mg^2+^, and Mn^2+^) in transformation. The addition of Mg^2+^and Mn^2+^did not improve the transformation level, in contrast to Ca^2+^, which, upon addition alone or combined with other divalent cations, slightly increased the transformation levels (2-fold) (see Fig. S5B in the supplemental material). Finally, we tested the effect of the agarose concentration in the transformation medium and observed that at 0.25 to 1% agarose, bacteria were increasingly and steadily transformable (by 2- to 3-fold in comparison to the control condition 0.5%) (Fig. 5B).

Altogether, using flow cytometry, we refined the optimal conditions for the transformation of A. baumannii strain AB5075 and improved by 100-fold the transformation frequencies of this strain (compare Fig. 3 to 5B).

### Probing natural transformation in nonclinical and MDR clinical animal isolates.

**FIG 6:**
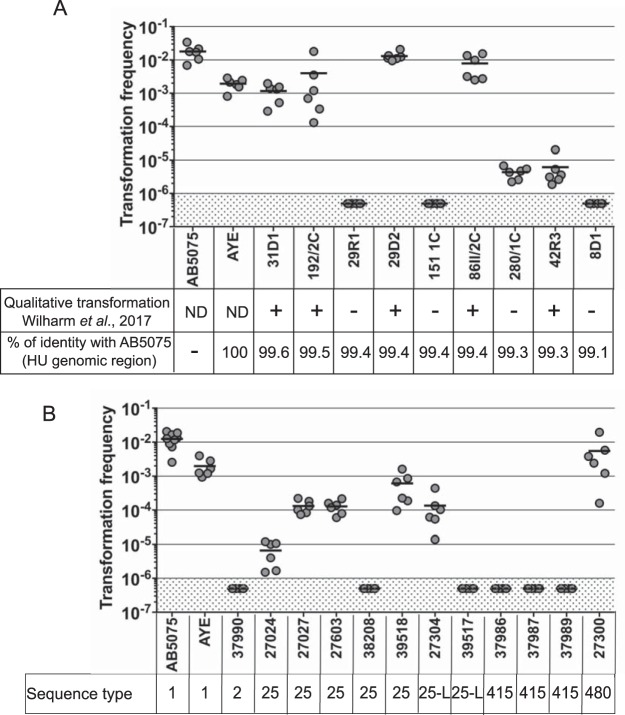
Determination of natural transformation frequencies of nonclinical and clinical A. baumannii isolates using flow cytometry. Wild animal (A) and clinical animal (B) isolates of A. baumannii were subjected to transformation using pASG-5 and the optimized conditions developed for the strain. (A) Results of transformation ability obtained in a qualitative manner are reproduced from the work of Wilharm et al. (33). ND, not done; +, transformable; –, not transformable. The percent sequence identity between the HU region of strain AB5075 (4,220 nucleotides long) with the recipient strain is indicated. (B) Sequence types (Pasteur scheme) of clinical isolates are specified (with 25-L representing ST25-like). A total of six to nine independent transformation assays were performed on two or three separate occasions. The horizontal lines represent the mean measurements for each strain. The limit of detection (10^−6^) is indicated by shading.

To further validate the cytometry-based method and to gain insight into the transformation ability of nonclinical strains isolated from wild animals, we sought to estimate the transformation ability of nine fully sequenced strains isolated from wild storks, some of which were related to human clinical isolates (33). Taking advantage of avian strain genome availability (33), we confirmed that the HU gene and the flanking regions are highly conserved with at least 99.1% identity between the avian strain sequences and the AB5075 strain sequence. Previously, five of these nine strains were found to be transformable in a qualitative transformation assay using the antibiotic selection-based method (33). All five strains were also transformable using the fluorescence-based method, but their ability to undergo transformation spreads over a range of 3 orders of magnitude (Fig. 6A). Remarkably, the fluorescence based-method allowed us to detect transformation in one isolate that was not identified as transformable using the antibiotic selection method (33) (Fig. 6A, strain 280/1C). There is no clear correlation between the transformation level and the sequence identity of the transforming sequence given that strains that present the same sequence identity (99.4%) (Fig. 6A) are either highly transformable (strains 29D2 and 86II/2C) or not transformable (strains 29R1 and 151/1C). Remarkably, strains presenting levels sequence identities lower than the strain AYE (strains 29D2, 192/2C, and 86II/2C) still presented comparable levels of transformation (Fig. 6A).

Taking advantage of the flow cytometry-based method and improved conditions for testing transformation, we then investigated the transformation profile of clinical MDR A. baumannii isolates, again of animal origin (typed but not fully sequenced). We estimated the transformation frequencies of 12 A. baumannii isolates from diseased animals obtained through the French Surveillance Network for Antimicrobial Resistance in Animal Pathogens (Resapath). These isolates presented multiple antibiotic resistance, including to carbapenems (34). For 50% of the isolates (6 of 12) transformation could be detected, with transformation frequencies ranging from 10^−6^, the limit of detection, up to 10^−2^ (Fig. 6B). Interestingly, most sequence type 25 (ST25) and ST25-like isolates were found to be transformable (5 of 7), in contrast to all of the ST415 isolates (*n* = 3). Altogether, these results on animal clinical isolates and nonclinical isolates demonstrate that the fluorescence-based method allows the detection of transformation events in A. baumannii regardless of the origin of the strain.

## DISCUSSION

Acinetobacter baumannii is now an established member of the growing list of bacteria capable of undergoing natural genetic transformation (13, 33). Together with genomic evidence of high recombination rates and multiple horizontal gene transfer events, natural transformation should be considered a major contributor of antibiotic resistance genes acquisition in A. baumannii. To investigate natural transformation in clinical MDR isolates, we developed here a fluorescence-based assay to detect and quantify transformation. To select an optimal marker of transformation, we engineered seven chromosomal markers consisting in C-terminal translational fusion of sfGFP with abundant proteins. Interestingly, we obtained an sfGFP fusion protein with the Fis protein, an essential protein in AB5075 (35). However, the level of expression of this chimeric protein did not confer the cells a fluorescence level distinguishable from autofluorescence (Fig. 1B and C; see also Fig. S1 in the supplemental material). All other translational fusions were highly expressed and emitted a fluorescence signal that could distinguish the corresponding strains from the wild-type strain (Fig. 1; see also Fig. S1 in the supplemental material). Expectedly, the fluorescence intensity of the cells correlates with abundance of the sfGFP fusion protein (see Fig. S1 in the supplemental material). Yet, protein folding or its location in the bacterial cell also influences the fluorescence intensity of a protein fusion. For instance, HU-sfGFP and HNS-sfGFP fusions are both predicted to be associated with the nucleoid and seemed to be expressed at similar levels (see Fig. S1 in the supplemental material). However, the HU-sfGFP fusion was brighter than the HNS one regardless of the growth phase (Fig. 1 and S2). While the HU-sfGFP signal appears distributed evenly in the cell (Fig. 1B), the HNS-sfGFP signal was restricted to one to two discrete foci per cell. Both subcellular localizations observed in A. baumannii were reminiscent of localization patterns observed for HU and H-NS in Escherichia coli (36). Besides their use as fluorescent markers of natural transformation, the fusion proteins could be useful to further investigate the function of nucleoid-associated proteins in A. baumannii. However, the functionality of the fusion protein was not assessed in this study given that HU and HNS, along with RpsA and DnaK, are nonessential proteins for growth in rich medium (35).

The HU-sfGFP marker combined with flow cytometry performed at least as well as the classical antibiotic selection to determine transformation frequencies (Fig. 2 and 3). We thus investigated the chemical and physical parameters susceptible to alter transformation efficiencies in A. baumannii. We tested the influence of divalent cations Mg^2+^, Ca^2+^, and Mn^2+^ on transformation efficiency to find a mild (2-fold) but significant effect of Ca^2+^, as in A. baylyi and A118. In A118, BSA was found to increase transformation by 2-fold (37), and yet the addition of this blood protein to the transformation medium results in a slight decrease in transformation efficiency in AB5075 (see Fig. S5A in the supplemental material). With a difference greater than 2 orders of magnitude, the parameter influencing transformation the most is pH (Fig. 5B). We found that transformation is more efficient at slightly acidic pH (5, 6), which is opposite to results obtained in A. baylyi ADP1 for which transformation drops by 10 to 100-fold at pH below neutrality (31). This is a clear indication that even closely related species can undergo natural transformation under specific conditions. Transformation occurring at mildly acidic pH in AB5075 led us to hypothesize that competence may be triggered in several specific sites within a host. For instance, acidic pH values are reminiscent of cutaneous pH (which is slightly lower than 5 [38]), or parts of the gastrointestinal tract, with pH levels of 5 to 7 in the colon (39). Noteworthy, these body sites contain large bacterial communities, including A. baumannii or other Acinetobacter species, that could offer substrates for natural transformation and foster the acquisition of antibiotic resistance genes (4, 5).

We also found that increasing agarose concentration improved transformation (Fig. 5A). A simple explanation for the apparent agarose concentration-dependent increase of natural transformation could be that at high concentrations, agarose physically impairs bacterial movement and somehow increases the local DNA/bacterial cell ratio and therefore transformation efficiency. Another hypothesis could be that mechanochemical perception of type IV pilus retraction on agarose stimulates competence. Indeed, sensing pilus retraction was involved in the induction of virulence gene expression and holdfast synthesis, respectively, in Pseudomonas aeruginosa and Caulobacter crescentus (40, 41). Also, and intriguingly, agarose is a necessary component of the semisolid media required to natural transform A. baumannii or closely related species (13, 33, 42). One hypothesis could be that the competent state for natural transformation is induced by the presence of agarose itself. Indeed, agarose, a polymer produced by seaweed, is made of repeating units of disaccharide (d-galactose and 3,6-anhydro-l-galactopyranose). Some soil or marine isolates of Acinetobacter sp. produce an agarase able to degrade agarose (43, 44). Similarly to the degradation products of chitin that induce competence in Vibrio cholerae (45), the degradation products of agarose could represent the trigger of competence in A. baumannii. The regulatory cascade that triggers competence in A. baumannii is currently unknown, and its identification may provide clues about the inducing cues.

Regardless of the inducing mechanism, the improved assay results in the highest transformation frequencies ever reported for A. baumannii, which can be as high as 1% for strain AB5075. The combined improved assay and method of detection made possible the investigation of natural transformability on a panel of nonclinical strains and clinical isolates. We found that 50% of A. baumannii clinical isolates isolated from pets (6 of 12) and 66% of nonclinical strains isolated from wild birds (6 of 9) were transformable. Among transformable strains or isolates, the transformation levels vary from 10^−2^ down to the detection limit (10^−6^). The variability of transformation efficiencies among A. baumannii isolates is reminiscent of the transformation levels observed in A. baumannii human clinical isolates (13) but also among isolates of other transformable species, such as S. pneumoniae or Haemophilus influenzae (46, 47). We ruled out the hypothesis that HU genomic region divergence prevents transformation. However, although HU gene and protein sequences are highly conserved, the expression level of the HU gene in the recipient strain varies, with some strains expressing less of the HU gene under the condition tested, resulting in cells presenting a lower fluorescent signal (see Fig. S6 in the supplemental material). Therefore, although the strain may be transformable, it is possible that transformants escape detection. As discussed beforehand, another explanation for the undetectable transformability may be related to strain-specific requirements for competence development. In this regard, the fact that most of the ST25 strains presented high level of transformation indicates that competence development is favored by the experimental conditions set for ST1 strains (AB5075 and AYE). These conditions may not be optimal for some strains, and we cannot rule out the possibility that strains that appear nontransformable in our assay would someday reveal themselves to be transformable under slightly different conditions. Variability among A. baumannii isolates may also result from mechanisms limiting transformation, such as secreted endonucleases that inhibit transformation in Campylobacter jejuni and V. cholerae (48–50).

A major limitation of our assay lies in its relative low sensitivity. Using our current equipment and settings, our assay does not allow us draw conclusions about the transformability of strains that would transform at frequencies below 10^−6^. Yet, the use of HU-sfGFP and flow cytometry offers, for now, the possibility to phenotypically detect transformation events in clinical isolates of A. baumannii for which transformability could not have been otherwise investigated. Little is known about the conditions that foster horizontal gene transfer by transformation in A. baumannii. Our easy and rapid method now allows study of the environmental conditions that may influence the rates of horizontal gene transfer using undomesticated strains. For instance, the method could be used to quantify horizontal gene transfer by natural transformation resulting from intra- or interspecific predatory behaviors (50, 51). The new HU-sfGFP would also constitute an excellent tool for the live-cell imaging of transformation in this species. It also opens investigations on horizontal gene transfer in infection models, experimentally testing the possibility that conditions specific to body sites, exposure to antibiotics, or antiseptics stimulate horizontal gene transfer.

## MATERIALS AND METHODS

### Bacterial strains, typing, and growth conditions.

The bacterial strains or strains used in this study are listed in Table S1 in the supplemental material. Genotyping of the isolates was based on sequence type determination according to the multilocus sequence typing method described previously (52). Unless specified otherwise, Acinetobacter baumannii isolates and strains were grown in lysogeny broth (LB; Lennox). All experiments were performed at 37°C.

### Construction of bacterial strains and plasmids.

All the oligonucleotides used in this study for genetic modification are listed in Table S2 in the supplemental material. Plasmid pASG-1 was constructed by cloning chimeric PCR product from assembly of PCR1 (primers asg-2 and asg-4 on pKD-sfGFP were obtained from Erwan Gueguen [Université de Lyon]) and PCR2 (primers Apr_Fw3 and asg-3 on plasmid carrying an apramycin resistance cassette) into pX5, a pMMB207 derivative to place the sfGFP gene under the control of an artificial strong constitutive promoter. The plasmid pASG-5 was constructed by cloning a PCR performed on genomic DNA extracted from AB5075 *hu-sfGFP* strain using the primers mlo-32 and mlo-35 into pJET1.2 according to the manufacturer's instructions (CloneJET PCR cloning kit; Thermo Fisher Scientific). Plasmid pMHL-2 is a pGEM-T Easy derivative in which an apramycin resistance cassette and a *sacB* counterselection cassette have been cloned. All plasmid sequences are available upon request.

Gene disruptions were performed using overlap extension PCR to synthesize a large chimeric DNA fragment carrying the selection/detection marker flanked by 2-kb fragments that are homologous to the insertion site. The oligonucleotides used for strain construction are listed in Table S2 in the supplemental material. Briefly, the coding sequence of the GFP superfolder (sfGFP) and the apramycin resistance cassette [*aac*(*3*)*IV*] were amplified from plasmid pASG-1 using the primers mlo-28 and mlo-29. The DNA fragments allowing homologous recombination (2 kb upstream and downstream of the targeted locus) were obtained by PCR on genomic DNA of strain AB5075. In a second step, the fragments previously obtained were assembled by PCR. The PCRs were performed with a high-fidelity DNA polymerase (PrimeStarMax; TaKaRa). Subsequently, each PCR fragment was purified after migration on an agarose gel, followed by extraction as recommended by the manufacturer (QIAquick gel extraction kit; Qiagen). For fusion between the nucleoproteins and sfGFP, a linker sequence that encodes the RGSGGEAAAKAGTS sequence between sfGFP and the nucleoprotein has been added so as not to disturb the functioning of the proteins (26). All chimeric PCR products were independently introduced into the AB5075 wild-type strain using natural transformation as described above.

To obtain a chromosomal marker without antibiotic cassette (*hu-sfgfp* strain), we first inserted a *sacB-aac* cassette (amplified from pMHL-2 plasmid) into the *hu* gene using overlap extension PCR. The resulting strain was subsequently transformed with a chimeric PCR product carrying a *hu-sfgfp* fusion without an antibiotic determinant and counterselected recombinants on minimal medium (M63) with 10% sucrose.

### Fluorescence microscopy.

Five hundred microliters of culture was incubated 30 min at room temperature with formaldehyde (final concentration, 3.7%), DAPI (4′,6′-diamidino-2-phenylindole; final concentration, 3 μM), and FM4-64 (final concentration, 10 μg/ml). Then, 3-μl portions of the preparation were spotted onto a poly-l-lysine-coated coverslip. After adhesion of the bacterial cells, the coverslips were washed twice with phosphate-buffered saline (PBS). The specimens were then observed under a fluorescence microscope by an immersion objective lens for a final magnification of ×1,000 (EVOS FL; Life Technologies).

### Flow cytometry.

Bacterial suspensions were fixed with formaldehyde (final concentration, 3.7%) and membrane stained with FM4-64 (final concentration, 10 μg/ml) for 30 min at room temperature and then washed twice with PBS and further diluted in PBS to obtain about 10^6^ cells/ml. An Attune acoustic focusing cytometer (Life Technologies) was used for all flow cytometry acquisitions. Samples were run at a collection rate of 25 μl/min, and fluorescence emission was detected using a 530/30 bandpass filter for GFP fluorescence and a 640 long-pass filter for FM4-64 fluorescence. To avoid aggregates, a maximum concentration of 10^6^/ml FM4-64-positive particles was analyzed. Flow cytometry data were analyzed using Attune software. Green fluorescence was analyzed on a minimum of 20 million FM4-64-positive particles.

### Transformation assay.

The following method was adapted from one published previously (13). Overnight cultures at 37°C in LB liquid medium were diluted in PBS to obtain 10^7^ CFU/ml. Then, 10 μl of bacterial suspension was mixed with 10 μl of substrate DNA (at 100 ng/μl for genomic DNA), and 2.5 μl of this bacterial/DNA suspension was then stabbed into 1 ml of freshly prepared motility medium (5 g/liter agarose, 5 g/liter tryptone, and 2.5 g/liter NaCl) in 2-ml Eppendorf tubes. After 20 h at 37°C, 200 μl of PBS was added to the tube, and the bacteria were resuspended by vigorous vortexing. The transformants were then selected by plating on selective agar media (apramycin, 30 μg/μl), or their fluorescence was measured by flow cytometry. Transformation frequencies were then determined by calculating the ratio of the number of transformant CFU (apramycin resistant or fluorescent) to the total number of CFU (antibiotic sensitive or nonfluorescent).

All of the transformation assays were performed on two or three separate occasions. On each occasion, three independent transformation reactions were conducted (three different bacterial cultures and DNA samples). All of the independent data points and the means of the measurements are plotted. Since normality of the distribution of transformation frequency does not apply for transformation frequency analysis, only nonparametric tests were performed (Mann-Whitney-Wilcoxon and Spearman correlation tests).

## Supplementary Material

Supplemental file 1
