# Supplementary material for: Fluorescence-Based Detection of Natural Transformation in Drug-Resistant Acinetobacter baumannii
Source: J Bacteriol. 2018 Sep 10;200(19):e00181-18. doi: 10.1128/JB.00181-18 (PMC6148472; doi:10.1128/JB.00181-18)
Supplement: Supplemental file 1 [file zjb999094872s1.pdf]

Fluorescence-based detection of natural transformation in drug resistant *Acinetobacter baumannii*

Authors: Anne-Sophie Godeux[a,b], Agnese Lupo[c], Marisa Haenni[c], Simon Guette-Marquet[a], Gottfried Wilharm[d], Maria-Halima Laaberki[a,b,#,*] and Xavier Charpentier[a,#,*].

[a] CIRI, Centre International de Recherche en Infectiologie, Inserm, U1111, Université Claude Bernard Lyon 1, CNRS, UMR5308, École Normale Supérieure de Lyon, Univ Lyon, 69100, Villeurbanne, France
[b] Université de Lyon, VetAgro Sup, 69280 Marcy l'Etoile, France.
[c] Unité Antibiorésistance et Virulence Bactériennes, Université Claude Bernard Lyon 1, ANSES Site de Lyon, Lyon, France
[d] Robert Koch Institute, Wernigerode Branch, Wernigerode, Germany.
[#] Contributed equally
* Correspondence to Maria-Halima Laaberki, maria-halima.laaberki@vetagro-sup.fr and Xavier Charpentier, xavier.charpentier@univ-lyon1.fr.

Supplementary material

Table S1. Bacterial strains used in this study

Strain	Genotype/comment	Reference or source
A. baumannii AB5075	wild type	(1)
A. baumannii AB5075	*cysI::Pst_sfGFP-aac*	this study
A. baumannii AB5075	*hu_sfGFP-aac*	this study
A. baumannii AB5075	*hns_sfGFP-aac*	this study
A. baumannii AB5075	*fis_sfGFP-aac*	this study
A. baumannii AB5075	*dnaK_sfGFP-aac*	this study
A. baumannii AB5075	*rpsA_sfGFP-aac*	this study
A. baumannii AB5075	*hu_sfGFP*	this study
A. baumannii isolate 27024	MDR isolate from RESAPTH	this study
A. baumannii isolate 27027	MDR isolate from RESAPTH	this study
A. baumannii isolate 27300	MDR isolate from RESAPTH	this study
A. baumannii isolate 27304	MDR isolate from RESAPTH	this study
A. baumannii isolate 27603	MDR isolate from RESAPTH	this study
A. baumannii isolate 37986	MDR isolate from RESAPTH	this study
A. baumannii isolate 37987	MDR isolate from RESAPTH	this study
A. baumannii isolate 37989	MDR isolate from RESAPTH	this study
A. baumannii isolate 37990	MDR isolate from RESAPTH	this study
A. baumannii isolate 38208	MDR isolate from RESAPTH	this study
A. baumannii isolate 39517	MDR isolate from RESAPTH	this study
A. baumannii isolate 39518	MDR isolate from RESAPTH	this study
A. baumannii strain 86II/2C	Avian non-clinical strain, white stork	(2)
A. baumannii strain 192/2	Avian non-clinical strain, white stork	(2)
A. baumannii strain 29D2	Avian non-clinical strain, white stork	(2)
A. baumannii strain 31D1	Avian non-clinical strain, white stork	(2)
A. baumannii strain 42R3	Avian non-clinical strain, white stork	(2)
A. baumannii strain 8D1	Avian non-clinical strain, white stork	(2)
A. baumannii strain 29R1	Avian non-clinical strain, white stork	(2)
A. baumannii strain 280/1C	Avian non-clinical strain, white stork	(2)
A. baumannii strain 151/1C	Avian non-clinical strain, white stork	(2)

Table S2. Oligonucleotides used in this study for DNA manipulation (plasmid construction and overlapping extension PCR)

Genetic construction	Name	Sequence 5' to 3'	Template; Annealing site
Chromosomal modifications using overlap extension PCR			
cysI::Pst-sfgfp_aac	mlo-20	tccaggtggttataaccc	AB5075 genomic DNA; 2kb upstream of the *cysI* gene
	mlo-21	ccaccatcaaacaggattttcgccgcaactacacctgcatactgg	AB5075 genomic DNA; 5' of the *cysI* gene
	mlo-22	ggcgaaaatcctgtttgatggtgg	pASG-1; upstream of Pst-*sfgfp-aac* for PCR fusion
	mlo-23	tgtcgactcatgagctcagccaatcgactgg	pASG-1; downstream of Pst-*sfgfp-aac* for PCR fusion
	mlo-24	ccagtcgattggctgagctcatgagtcgacataatgaagcaggcgagatgggc	AB5075 genomic DNA; 3' of the *cysI* gene
	mlo-25	ccaatcccaatccatcctgc	AB5075 genomic DNA; 2kb downstream of the *cysI* gene
sfgfp_aac marker	mlo-28	gcggcgaagcggcggcgaaagcgggcaccagctctaaaggtgaagaactgttcaccgg	pASG1; sfGFP (with linker)
	mlo-29	tcatgagctcagccaatcgactgg	pASG1; end of the ApraR cassette
hns-sfgfp_aac	mlo-26	ttacccgatgcaactagcc	AB5075 genomic DNA; 2kb upstream of the *hns* gene
	mlo-27	gctttcgccgccgcttcgccgccgctgccgcggattaagaaatcttcaagttttgcacc	AB5075 genomic DNA; 3' of *hns* gene for N-ter fusion (linker)
	mlo-30	ccagtcgattggctgagctcatgagcctcttaaaaagccaagcg	AB5075 genomic DNA; *hns* intergenic region
	mlo-31	tcttccatgtttgggcatagg	AB5075 genomic DNA; 2kb downstream of the *hns* gene
hu-sfgfp_aac	mlo-32	tttgaacgttgtctcggc	AB5075 genomic DNA; 2kb upstream of the *hu* gene
	mlo-33	gctttcgccgccgcttcgccgccgctgccgcgagcaactgaatctttaagacctttacc	AB5075 genomic DNA; 3' of *hu* gene for N-ter fusion (linker)
	mlo-34	ccagtcgattggctgagctcatgataaacgcgccaatataggc	AB5075 genomic DNA; *hu* intergenic region
	mlo-35	tgttgaccacgagattggcgg	AB5075 genomic DNA; 2kb downstream of the *hu* gene
dnaK-sfgfp_aac	mlo-57	taggtactaccaactcatgtg	AB5075 genomic DNA; 2kb upstream of the *dnaK* gene
	mlo-58	gctttcgccgccgcttcgccgccgctgccgcgttttttgtcatcttttacttcagtg	AB5075 genomic DNA; 3' of *dnaK* gene for N-ter fusion (linker)
	mlo-59	ccagtcgattggctgagctcatgaagaccgcgcgaaagcgc	AB5075 genomic DNA; *dnaK* intergenic region
	mlo-60	aatacatcagcttgggc	AB5075 genomic DNA; 2kb downstream of the *dnaK* gene
rpsA-sfgfp_aac	mlo-62	ctgcttcagctgagtcgcg	AB5075 genomic DNA; 2kb upstream of the *rpsA* gene
	mlo-63	gctttcgccgccgcttcgccgccgctgccgcgcttcatttgtgctttgatcaagtcacc	AB5075 genomic DNA; 3' of *rpsA* gene for N-ter fusion (linker)
	mlo-64	ccagtcgattggctgagctcatgaactgtaaacggtagcg	AB5075 genomic DNA; *rpsA* intergenic region
	mlo-65	gagttaggtgggattgc	AB5075 genomic DNA; 2kb downstream of the *rpsA* gene
fis-sfgfp_aac	mlo-67	ccagatgtgccgatgcgtc	AB5075 genomic DNA; 2kb upstream of the *fis* gene

	mlo-68	gctttcgccgccgcttcgccgccgctgccgcgttcactcattaaaccgtgagc	AB5075 genomic DNA; 3’ of *fis* gene for N-ter fusion (linker)
	mlo-69	ccagtcgattggctgagctcatgaaatgctcacggcttcg	AB5075 genomic DNA; *fis* intergenic region
	mlo-70	cgcctgcttcacgcgcagc	AB5075 genomic DNA; 2kb downstream of the *fis* gene
hu::sacB_aac	mlo-32	tttgaacgttgtctcggc	AB5075 genomic DNA; 2kb upstream of the *hu* gene
	asg-57	cggcaggtatatgtgatgggtgatagattaagcaactgaatctttaagacc	AB5075 genomic DNA; 3’ of *hu* gene
	mlo-29	tcatgagctcagccaatcgactgg	pMHL-2; *aac* gene
	asg-58	atcacccatcacatatacctgccg	pMHL-2; *sacB* gene (chimeric primer)
	mlo-34	ccagtcgattggctgagctcatgataaacgcgccaatataggc	AB5075 genomic DNA; *hu* intergenic region
	mlo-35	tgttgaccacgagattggcgg	AB5075 genomic DNA; 2kb downstream of the *hu* gene
hu-sfgfp	mlo-32	tttgaacgttgtctcggc	AB5075 *hu-sfGFP_aac* genomic DNA;<br>2kb upstream of the *hu* gene
	asg-59	gaagcagctccagcctacacaatc	AB5075 *hu-sfGFP_aac* genomic DNA;<br>*sfgfp* gene
	asg-60	gattgtgtaggctggagctgcttctaaacgcgccaatataggc	AB5075 *hu-sfGFP_aac* genomic DNA;<br>*sfgfp* gene and intergenic region downstream *hu* gene
	mlo-35	tgttgaccacgagattggcgg	AB5075 *hu-sfGFP_aac* genomic DNA; 2kb downstream of the *hu* gene
Primers for pASG-1 construction			
	asg-1	atatcatatgtctaaaggtgaagaactgttcaccgg	annealing on sfGFP gene, NdeI site
	asg-4	ggaacttcgaagcagctccagcctacacaatcttatttgtagagctcatccatgccgtgc	annealing downstream sfGFP gene
	Apr_Fw3	gattgtgtaggctggagctgcttcgaagttccatcaaggcccgatccttggagcccttg	annealing upstream of Apramycin resistance gene
	asg-3	atataaagcttgtcgactcatgagctcagccaatcgactgg	annealing downstream of Apramycin resistance gene, HindIII/SalI site

References

1. Jacobs AC, Thompson MG, Black CC, Kessler JL, Clark LP, McQueary CN, Gancz HY, Corey BW, Moon JK, Si Y, Owen MT, Hallock JD, Kwak YI, Summers A, Li CZ, Rasko DA, Penwell WF, Honnold CL, Wise MC, Waterman PE, Lesho EP, Stewart RL, Actis LA, Palys TJ, Craft DW, Zurawski DV. 2014. AB5075, a Highly Virulent Isolate of Acinetobacter baumannii, as a Model Strain for the Evaluation of Pathogenesis and Antimicrobial Treatments. mBio 5:e01076–14.

2. Wilharm G, Skiebe E, Higgins PG, Poppel MT, Blaschke U, Leser S, Heider C, Heindorf M, Brauner P, Jäckel U, Böhland K, Cuny C, Łopińska A, Kaminski P, Kasprzak M, Bochenski M, Ciebiera O, Tobółka M, Żołnierowicz KM, Siekiera J, Seifert H, Gagné S, Salcedo SP, Kaatz M, Layer F, Bender JK, Fuchs S, Semmler T, Pfeifer Y, Jerzak L. Relatedness of wildlife and livestock avian isolates of the nosocomial pathogen Acinetobacter baumannii to lineages spread in hospitals worldwide. Environ Microbiol 19:4349–4364.

Fluorescence-based detection of natural transformation in drug resistant *Acinetobacter baumannii*

Authors: Anne-Sophie Godeux[a,b], Agnese Lupo[c], Marisa Haenni[c], Simon Guette-Marquet[a], Gottfried Wilharm[d], Maria-Halima Laaberki[a,b,#,*] and Xavier Charpentier[a,#,*].

[a] CIRI, Centre International de Recherche en Infectiologie, Inserm, U1111, Université Claude Bernard Lyon 1, CNRS, UMR5308, École Normale Supérieure de Lyon, Univ Lyon, 69100, Villeurbanne, France
[b] Université de Lyon, VetAgro Sup, 69280 Marcy l'Etoile, France.
[c] Unité Antibiorésistance et Virulence Bactériennes, Université Claude Bernard Lyon 1, ANSES Site de Lyon, Lyon, France
[d] Robert Koch Institute, Wernigerode Branch, Wernigerode, Germany.
[#] Contributed equally
* Correspondence to Maria-Halima Laaberki, maria-halima.laaberki@vetagro-sup.fr and Xavier Charpentier, xavier.charpentier@univ-lyon1.fr.

Supplementary Figures

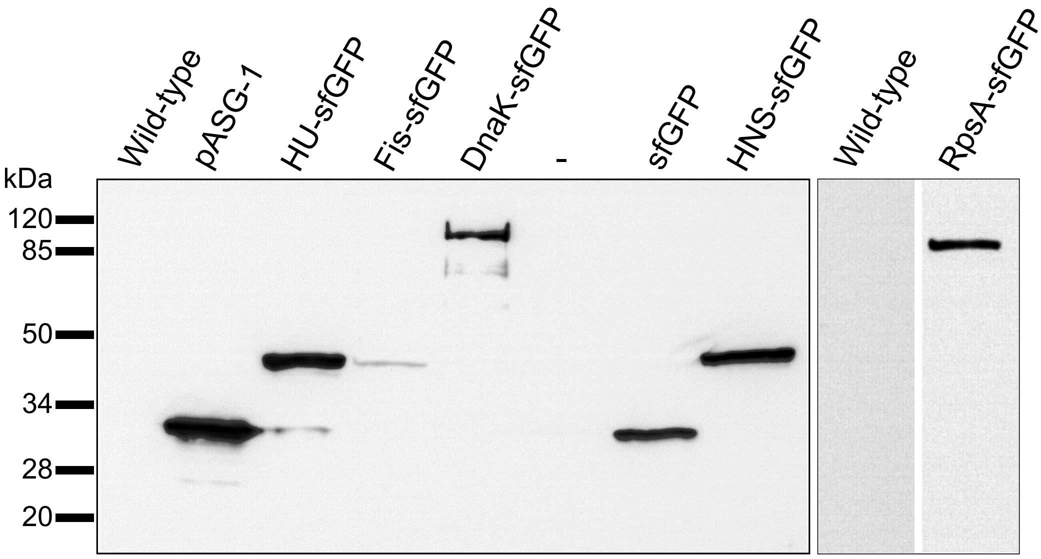

Figure S1. Immunoblotting performed on whole bacterial lysates from the various sfGFP constructs compared to the wild-type strain (WT).

Molecular mass standards are indicated in kilodaltons on the left side of the panel. Two independent western blots are delimited by black frames.

Each protein presented an apparent size corresponding to their theoretical molecular weight namely 27KDa for free sfGFP (expressed from a multicopy plasmid pASG-1), 37,2 KDa for HU-sfGFP, 38,2 KDa for Fis-sfGFP, 96,2 KDa for DnaK-sfGFP, 27KDa for free sfGFP (expressed from a neutral chromosomal locus, cysI), 40,7KDa for HNS-sfGFP, 89,2KDa for RpsA-sfGFP (MW of the chimeric proteins including the 10 amino-acid linker).

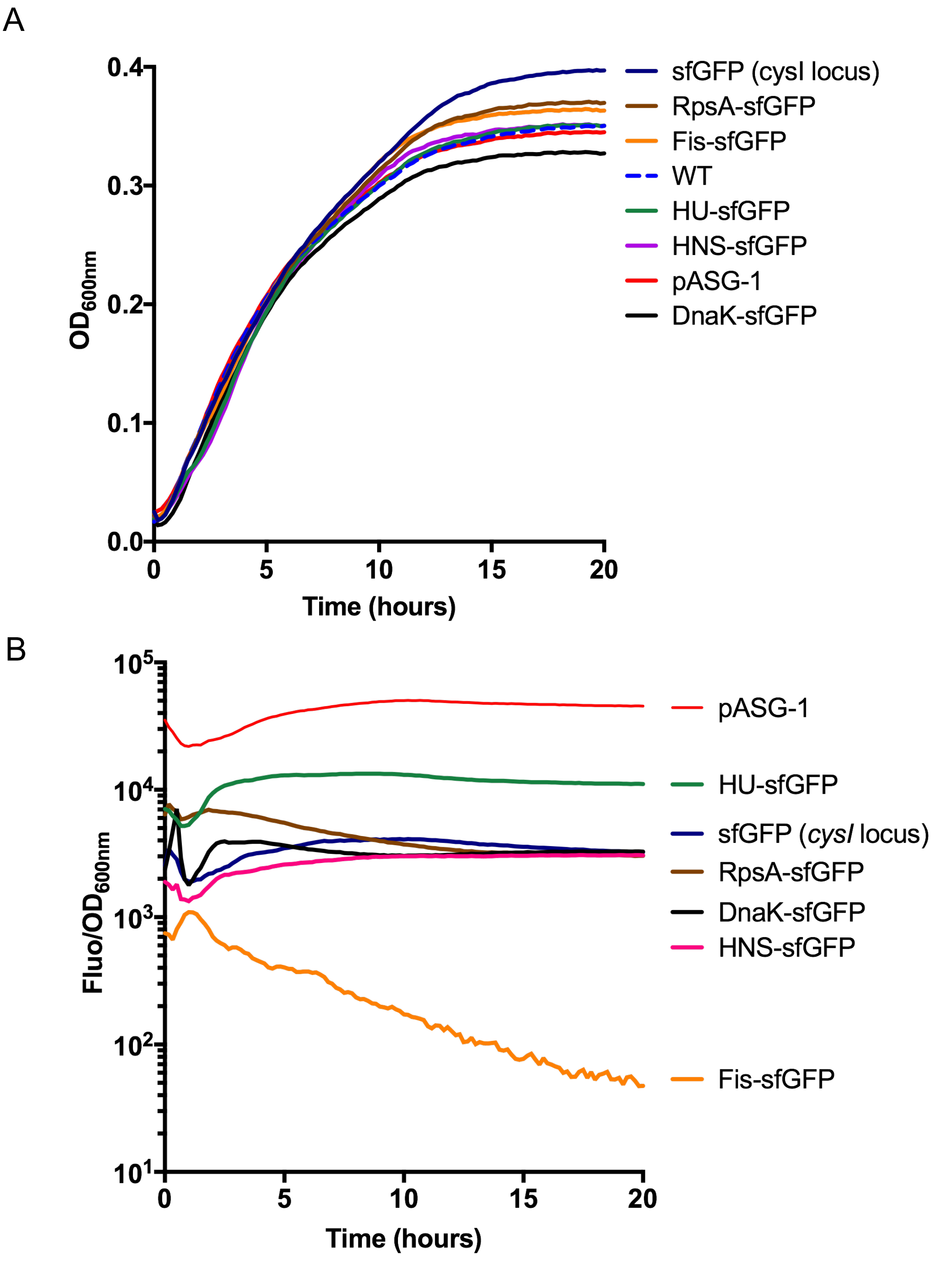

Figure S2. Growth and relative fluorescence during growth in liquid medium of AB5075 strain expressing various fluorescent markers

A. Bacterial growth curves throughout time of the seven fluorescent strains carrying either a multicopy plasmid (pASG-1) or chromosomal constructs (GFP expressed from a neutral locus (cysI), or GFP protein fusions) and of the wild type strain (WT). All strains were grown in tryptone-NaCl medium at 37°C.

B. Relative fluorescence to cell density of each strains (autofluorescence subtracted).

Green florescence and optical density were monitored using a 96-well plate reader.

A representative experiment is shown. For each graph, the legends are ordered from up to bottom as curves appear on the graphic.

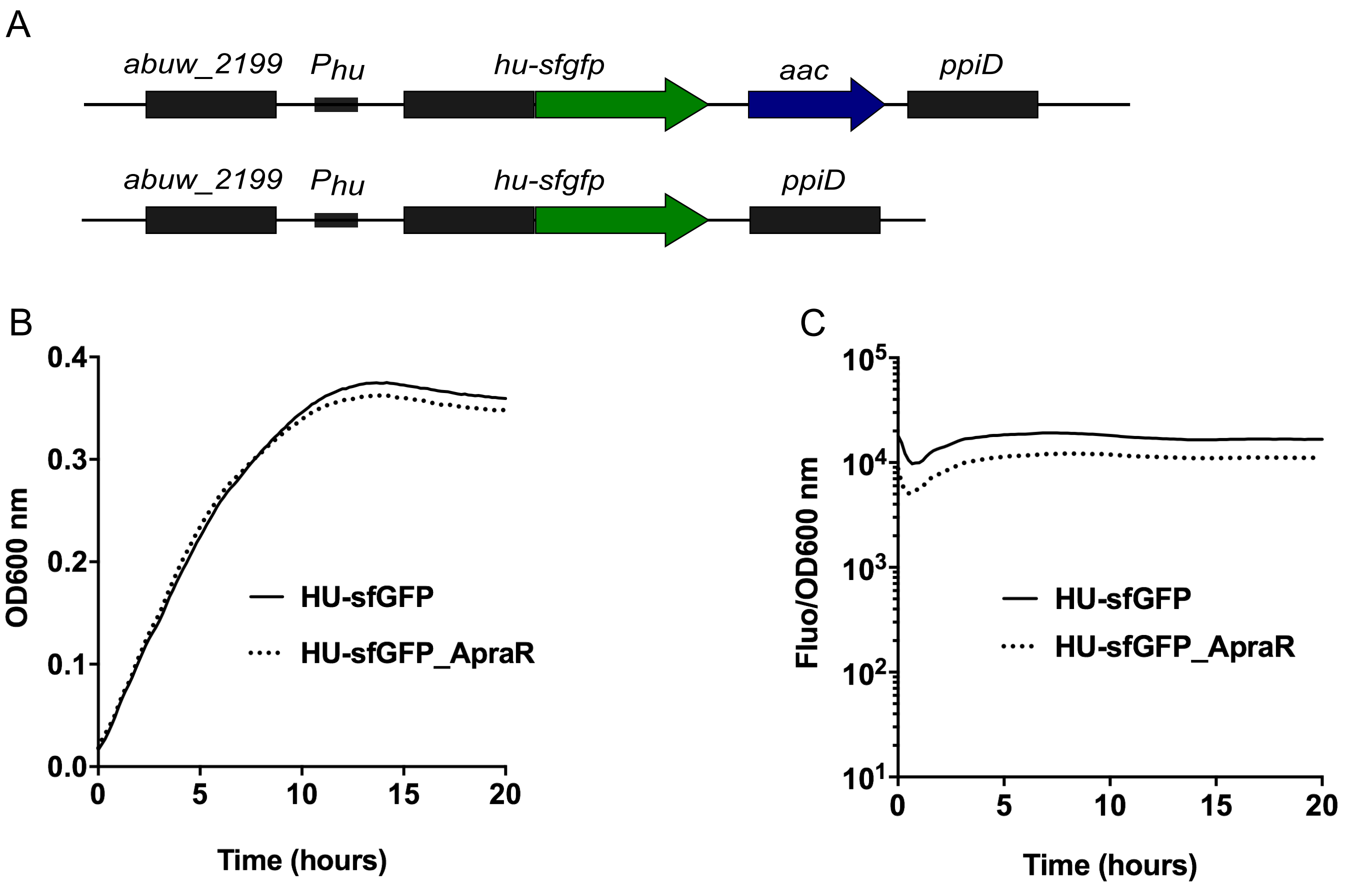

Figure S3. Validation of chromosomal HU-sfGFP marker without antibiotic resistance gene

A. Schematic representation of genetic constructions encoding for the *hu-sfgfp* markers with and without Apramycin resistance cassette (*aac*). The upstream and downstream genes of the hu gene are represented (respectively *abuw_2199* and *ppiD* genes).

Bacterial growth curves (B) and fluorescence relative to cell density (C) throughout time of both fluorescent strains grown in Tryptone-NaCl medium at 37°C. Bacterial green florescence and optical density (OD600nm) were monitored using a 96-well plate reader.

A representative experiment is shown.

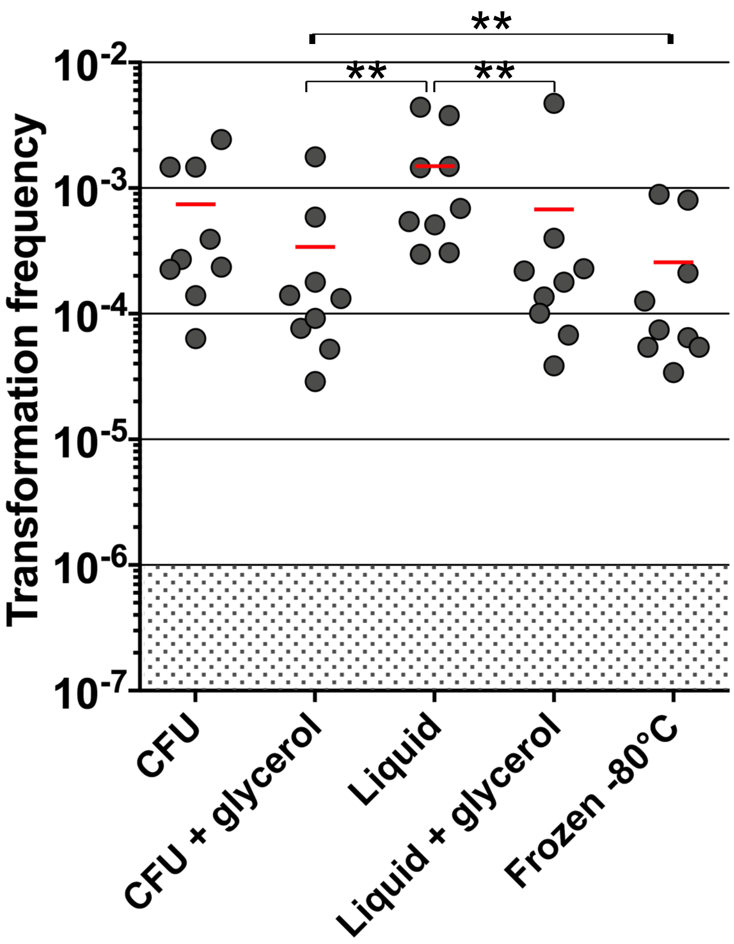

Figure S4. Effect of bacterial culture medium (agar or liquid) or storage (frozen) conditions prior to transformation.

- CFU: bacteria were grown on solid media (LB agar) and one colony forming unit (CFU) was resuspended in water prior to transformation assay.
- Liquid: bacteria were grown overnight in liquid medium (LB) prior to transformation.
- Frozen -80°C: after overnight growth in liquid LB, glycerol was added to a 30% final concentration and bacteria were stored at -80°C until resuspension in water prior to transformation assay.

When "+ glycerol" is indicated, glycerol was added to a final concentration of 30%.

A total of nine independent transformation assays were performed on three separate occasions. The horizontal lines represent means for each conditon. Comparison two by two using the non-parametric Mann-Whitney-Wilcoxon test (two tailed) between the various conditions gave *p*-values <0.01 (**). The limit of detection (10^{-6}) is indicated by a shaded grey area.

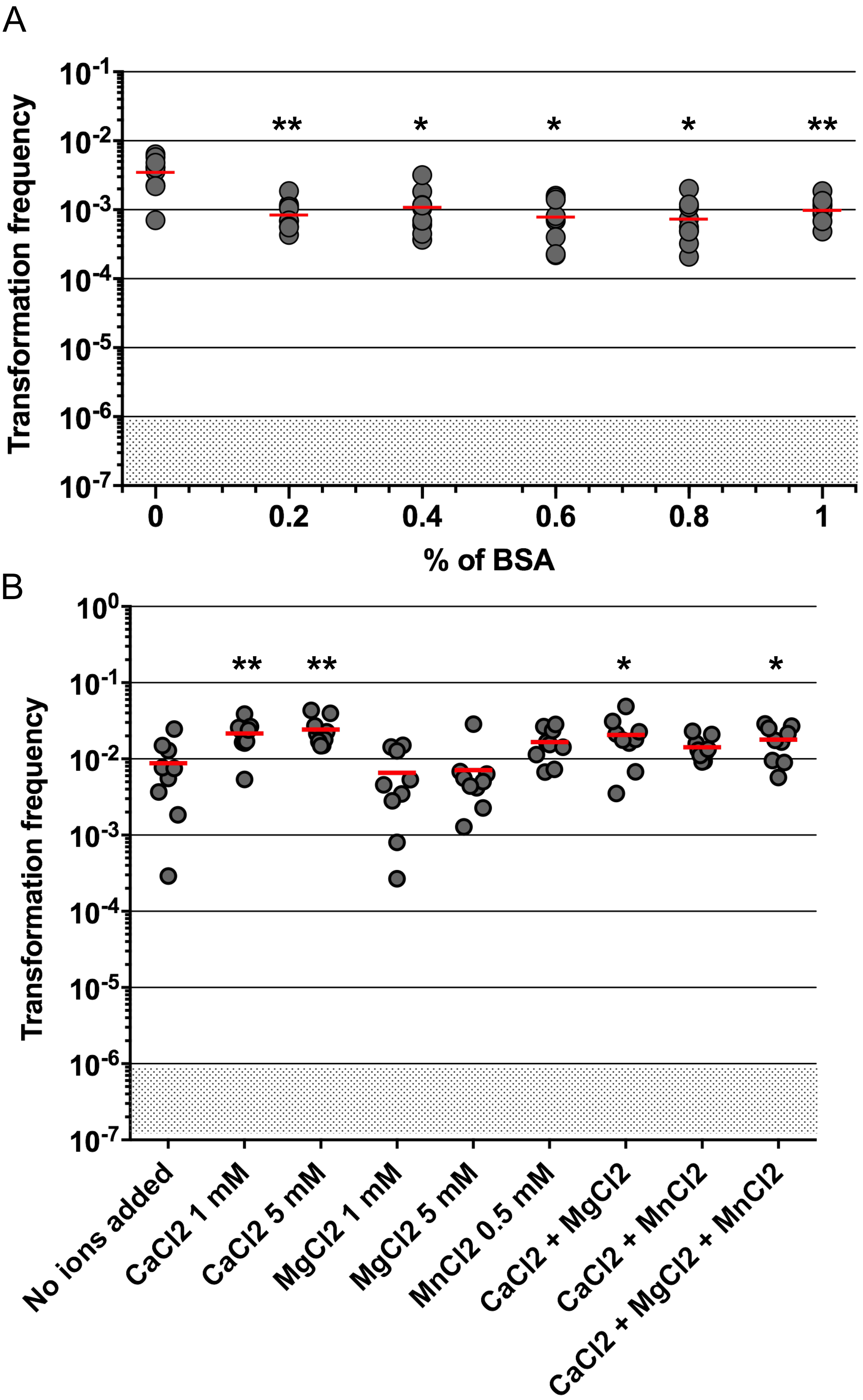

Figure S5. Effect of BSA and divalent cations on transformation

A. Effect of increasing concentrations of BSA on transformation efficiencies

B. Effect of divalent cations (Ca^{2+}, Mg^{2+}, Mn^{2+}) on transformation efficiencies. Concentration are indicated in mM.

A total of nine independent transformation assays were performed on three separate occasions.. The horizontal lines represent means for each conditon. The limit of detection (10^{-6}) is indicated by a shaded grey area.

Comparison to the control condition (no BSA or no added cations) using the non-parametric Mann-Whitney-Wilcoxon (two tailed) test gave *p*-values <0.01 (**) or <0.05 (*), other comparisons gave *p*-values >0.05.

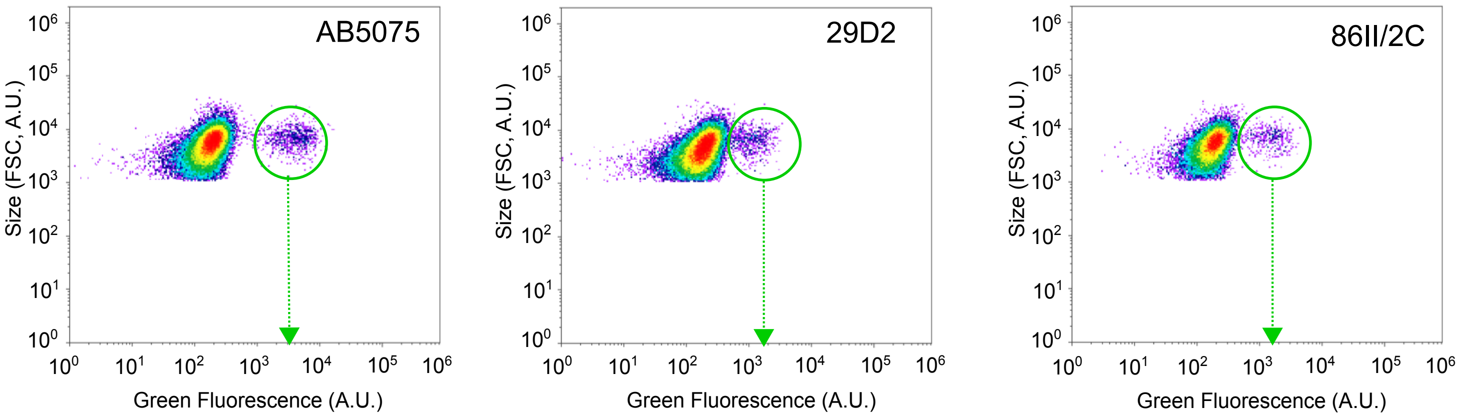

Figure S6. Intensity of green fluorescence in various strain background after transformation with HU-sfGFP marker.

Bacterial cells were recovered after transformation with pASG-5 plasmid of AB5075, 29D2 and 86II/2C strains and subjected to flow cytometry analysis. Density plots for green fluorescence intensity (abscissa) and size (FSC, ordinate) are shown (log scales). Among all the strains tested, transformed cells from strains 29D2 and 86II/2C presented a lower green fluorescence intensity in comparison to the reference strain (AB5075) as underlined by the green arrows. A representative experiment is shown.
